# The anticoagulation dilemma in post-operative atrial fibrillation after cardiac surgery

**DOI:** 10.1093/ehjcvp/pvag051

**Published:** 2026-07-07

**Authors:** Hanne Berg Ravn, Milan Milojevic

**Affiliations:** Department of Anesthesiology and Intensive Care, Odense University Hospital, J.B. Winsloews vej 4, Odense 5000, Denmark; Department of Clinical Research, Health Faculty, University of Southern Denmark, Odense DK-5000, Denmark; Department of Cardiac Surgery and Centre of Excellence, Dedinje Cardiovascular Institute, Belgrade 11040, Serbia; Department of Cardiac Surgery, University Hospital Zurich, University of Zurich, Zurich 8091, Switzerland

## Introduction

**Figure 1:**
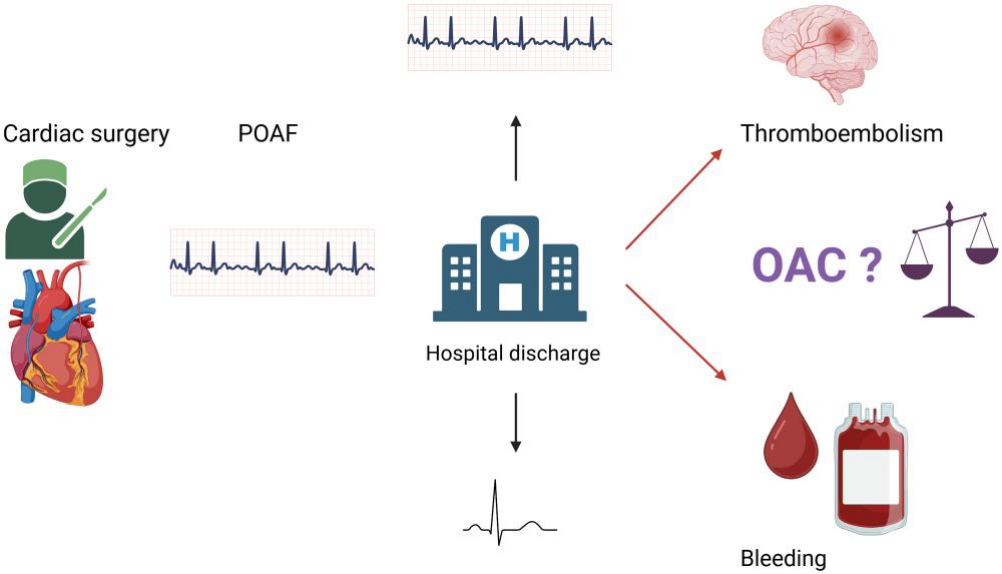
New onset post-operative atrial fibrillation is a frequent complication with prognostic importance following cardiac surgery. Despite its frequent occurrence, there is still limited evidence on how to assess the individual patient’s risk of thromboembolic- and bleeding events, when deciding on prescribing an oral anticoagulant. This paper summarizes the current knowledge and points out remaining gaps in the management of POAF after cardiac surgery.

Post-operative atrial fibrillation (POAF) is among the most frequent events following cardiac surgery. Its reported incidence varies substantially between 20% and 60%, depending on the type of surgery, patient characteristics, duration of rhythm monitoring, and study design.^1,2^ In-hospital electrocardiograms may fail to capture short-lasting or asymptomatic episodes compared with epicardial cardiac monitors, in which a substantially higher cumulative incidence of new-onset AF has been observed.^2,3^ (*Figure 1*).

Despite its frequency, the indication for oral anticoagulation (OAC) treatment after POAF remains one of the most debated topics in cardiac surgery. The central dilemma seems straightforward yet unresolved: on one side is the risk of thromboembolism, including stroke; on the other is the risk of anticoagulant-related bleeding. This uncertainty is amplified by the absence of well-designed randomized trials and by emerging recognition that POAF is a special post-operative entity, not simply the same condition as pre-existing atrial fibrillation (AFib).

## The burden of POAF: between transient arrhythmia and risk marker

Previously, POAF was considered a transient, self-limiting post-operative arrhythmia with limited long-term consequences. The perception has been challenged by accumulating observational data demonstrating both increased morbidity and mortality.^4^ This has led to new-onset AFib being frequently reported as a major secondary outcome in trials comparing surgery with interventional procedures, consistent with academic research consortium endpoint definitions that define it as an episode documented on a 12-lead ECG or lasting at least 30 s on rhythm monitoring.^5^ However, its interpretation requires caution. In contrast to irreversible and disabling clinical events, POAF is influenced by the intensity and duration of the arrhythmia and should not be equated with pre-existing AFib.

In a systematic review and meta-analysis including 246 340 patients, POAF was associated with increased perioperative mortality, stroke, myocardial infarction, and acute kidney injury, as well as long-term mortality, stroke, and recurrent AFib.^4^ Similarly, in the SWEDEHEART study of 24 523 patients undergoing first-time isolated CABG, POAF occurred in 30% of patients and was independently associated with ischaemic stroke, heart failure rehospitalisation, and recurrent AFib during a median follow-up of 4.5 years.^6^

Despite the strong association in observational studies, there is no evidence of POAF being the causation. POAF may simply be a marker of greater comorbidity, more complex surgery, inflammation, or underlying atrial vulnerability.

## The case for anticoagulation at discharge

The argument for OAC after POAF is based on the observation that POAF is associated with stroke and recurrent AFib, together with evidence from non-surgical AFib showing that anticoagulation reduces thromboembolic events in patients at elevated risk.

Guidelines reflect this uncertainty, too. The 2024 ESC AFib guidelines state that long-term OAC should be considered in high-risk patients with POAF after cardiac and non-cardiac surgery to prevent ischaemic stroke and thromboembolism (Class IIa, Level B).^7^ This recommendation is indeed difficult to translate into daily practice, since there is no definition of the required AF burden, the relevance of sinus rhythm at discharge, the optimal duration of anticoagulation, or how to estimate bleeding risk. The 2023 ACC/AHA/ACCP/HRS AF guideline is more prescriptive, stating that it is reasonable to administer anticoagulation for 60 days after surgery, with reassessment of the need for longer-term therapy.^8^

The 2024 EACTS perioperative medication guidelines are more specific for cardiac surgery. They recommend OAC for at least 4 weeks in patients with persistent POAF at discharge, followed by re-evaluation ∼1 month after surgery. In patients with POAF, who are in sinus rhythm at discharge, OAC for at least 4 weeks may be considered, taking bleeding and thromboembolic risks into account.^9^ This distinction is important: persistent POAF at discharge or transient POAF resolved before discharge should not be managed in the same way; rather, they should prompt an individualized assessment of rhythm status, thromboembolic and bleeding risk.

A further argument for OAC is the potential silent AFib recurrence after discharge. Without continuous monitoring, recurrent AFib episodes may occur undetected, leaving patients potentially unprotected for thromboembolism. However, whether such episodes are frequent, sustained, or cause thromboembolic events has only recently become clear.

## The case against anticoagulation at discharge

Despite a plausible rationale, evidence for the net clinical benefit of OAC after POAF remains inconsistent. In the SWEDEHEART CABG cohort, OAC initiation within 30 days of surgery after POAF was not associated with reduced ischaemic stroke or thromboembolism but with increased bleeding risk.^6^ An analysis from the Society of Thoracic Surgeons Adult Cardiac Surgery Database, including almost 39 000 patients with POAF after isolated CABG, found that OAC at discharge was associated with higher mortality, no reduction in ischaemic stroke, and increased bleeding after propensity matching.^10^

A recent meta-analysis of 28 observational studies by van Kar *et al*.,^11^ including more than 1.6 million patients undergoing isolated CABG, further challenged the routine use of OAC. Among patients with POAF, absolute event rates were low. Over a median follow-up of 4.6 years, POAF patients experienced 1.73 thromboembolic events, 3.39 deaths, and 2.00 bleeding events per 100 person-years compared with 1.14, 2.19, and 1.60 per 100 person-years in non-POAF patients. Interestingly, only 1 in 6 POAF patients received OAC. OAC was not associated with a significant reduction in thromboembolism or mortality, but with a significant increase in bleeding. These observational data cannot establish a treatment effect, and residual confounding remains possible. It is possible that clinicians preferentially anticoagulated patients perceived as having higher thromboembolic risk, thereby reducing the event rate to that of untreated patients. Nevertheless, the consistent bleeding signal, irrespective of OAC agent, is clinically important and cannot be ignored.

## New evidence: high incidence of AF, but low AF burden

One of the most informative studies is the CABG-AF study by Herrmann *et al*.^2^ In this prospective multicentre cohort study, 198 patients without previous arrhythmia received an insertable epicardial cardiac monitor during CABG, enabling continuous ECG monitoring for 1 year. New-onset AFib occurred in 48%, a higher rate than previously reported with standard monitoring. However, the median AFib burden during the first year was only 0.07% (IQR 0.02%–0.23%), corresponding to 370 min. AFib burden was concentrated in the first post-operative week: 3.65% on days 1–7, 0.04% on days 8–30, and essentially 0% from days 31–365. After discharge, only three patients had an AFib episode lasting longer than 24 h.

These findings are central to the anticoagulation debate, indicating that POAF should not be overestimated after CABG; it appears to be a transient condition with a very low long-term AFib burden. At the same time, it should not be underestimated, since it identifies patients at increased long-term risk of AFib recurrence and heart failure. The key question is therefore not whether POAF matters, but whether the magnitude and timing of AFib burden justify routine OAC in all patients after discharge.

## An unresolved dilemma: the path forward

Taken together, the available literature paints a conflicted but coherent picture. POAF is common and prognostically important, but OAC, whether with VKAs or DOACs, has not consistently translated these elevated risks into a lower number of thromboembolic events, while increasing bleeding risk. The missing evidence is a well-designed randomized trial, such as the ongoing PACeS trial (ClinicalTrials.gov: NCT04045665), comparing OAC vs. no OAC in carefully selected patients with POAF, with stratification according to rhythm status at discharge, AFib burden, stroke risk, bleeding risk, and concomitant antiplatelet therapy.

Until such evidence is available, a personalized, risk-stratified approach appears most defensible. The CHA_2_DS_2_-VASc score may help estimate thromboembolic risk, but the optimal threshold remains uncertain in patients with POAF.^12^ Equally important, bleeding risk must be assessed systematically. This is where the study by Rezk *et al*.^13^ represents an important step forward. Among 4436 patients with POAF after CABG who were not treated with OAC, the four-item PRECISE-DAPT score classified 36% as high bleeding risk. One-year major bleeding occurred in 0.8%, 1.9%, and 3.6% of low-, moderate-, and high-risk patients, respectively. High bleeding risk was associated with an almost five-fold higher hazard of major bleeding than low risk. Discrimination was moderate (AUC 0.68), and calibration was acceptable up to an annual bleeding risk of ∼7%. If OAC is selected, DOACs may represent a reasonable alternative to vitamin K antagonists in patients without mechanical heart valves or with moderate-to-severe mitral stenosis, although the evidence remains limited.^14^

The PRECISE-DAPT study should not be interpreted as proving that the algorithm should decide anticoagulation. Patients receiving OAC were excluded, bleeding events were limited to hospitalization or death, and the score was not developed specifically for POAF. However, it addresses a crucial gap acknowledged by guidelines: how to estimate bleeding risk when considering OAC after POAF.

The path forward should move beyond ‘anticoagulate all’ or ‘anticoagulate none’. Persistent POAF at discharge justify short-term OAC with reassessment. In patients restored to sinus rhythm before discharge and with a low AFib burden and high bleeding risk, rhythm monitoring or withholding OAC until AFib recurs may be reasonable. The studies above remind us that in POAF, the clinical question is not only who may have a stroke, but also who is likely to bleed.

## Data Availability

No new data are associated with this article. Figure preparation in BioRender.
